# Learning from Somaliland? Transferability of learning from volunteering to national health service practice in the UK

**DOI:** 10.1186/s12992-016-0146-z

**Published:** 2016-03-22

**Authors:** Esther Tillson, Sibylle Herzig van Wees, Charlotte McGowan, Hannah Franklin, Helena Jones, Patrick Bogue, Shirin Aliabadi, Paula Baraitser

**Affiliations:** Barts and the London Medical School, London, UK; King’s Centre for Global Health, London, UK; Hull York Medical School, London, UK; Kings Centre for Global Health, London, UK; University of Oxford, London, UK; Guy’s and St. Thomas’ NHS Trust, London, UK; King’s College London, London, UK

## Abstract

**Background:**

Capacity building partnerships between healthcare institutions have the potential to benefit both partners particularly in staff development. Previous research suggests that volunteering can contribute to professional development but there is little evidence on how learning is acquired, the barriers and facilitators to learning in this context or the process of translation of learning to the home environment.

**Results:**

Volunteers from a healthcare partnership between the UK and Somaliland reported learning in communication, interdisciplinary working, teaching, management, leadership and service development. This learning came from observing familiar practices in unfamiliar environments; alternative solutions to familiar problems; learning about Somali culture; opportunities to assume higher levels of responsibility and new professional relationships. There was variability in the extent of translation to NHS practice. Time and support available for reflection and mentoring were important facilitators of this process.

**Conclusions:**

The professional development outcomes documented in this study came directly from the experience of volunteering. Experiential learning theory suggests that this requires a complex process of critical reflection and new knowledge generation, testing and translation for use in new contexts. This process benefits from identification of learning as an important element of volunteering and support for reflection and the translation translation of learning to UK contexts. We suggest that missed opportunities for volunteer learning will remain until the volunteering process is overtly framed as part of continuing professional development.

## Background

Capacity building partnerships between healthcare institutions in high income countries and collaborating institutions in low or middle income countries have the potential to benefit both partners [1–3]. The work of these partnerships is often done by volunteer healthcare professionals and is thought to benefit both the individual volunteers through personal and professional development and the institutions where they work [1, 3]. Staff development is one reason that institutions in high income countries support involvement in links [4], and is an important element of the two-way transfer of knowledge that underpins the healthcare partnership concept [5].

A recent review of the literature identified possible benefits for UK healthcare professionals volunteering in partnerships that included clinical learning and the development of managerial, communication, teamwork and academic skills [6]. Longstaff identifies benefits to both the individual and the NHS using existing NHS frameworks for Continuing Professional Development [7]. The literature suggests that a wide range of skills can be attained but there is little evidence on how learning is acquired, the barriers and facilitators to learning in this context and the transferability of learning to work in high income countries. We aimed to address these questions by studying the learning process and transferability of learning for volunteers working within a long term healthcare partnership between Kings Health Partners (a UK academic health science centre), the Tropical Health & Education Trust (the UK support organisation for health links) and key healthcare educational institutions in Somaliland (Edna Adan Maternity Hospital, Boroma Group Hospital, and Boroma medical school).

### Kings—THET Somaliland partnership (KTSP)

KTSP was established in 2000 as a link between Kings College Hospital and two Somaliland hospitals, Edna Adan Maternity Hospital and Boroma Group Hospital, and Boroma Medical School. The link now also works with a further five medical teaching institutions in Somaliland.

Since 2000, 144 KTSP volunteers have provided support through short-term volunteer trips. Healthcare professionals, managers, academics and researchers have contributed to healthcare professional training and capacity building within the Ministry of Health and professional associations. The partnership is driven by the needs of healthcare institutions in Somaliland who identify areas where UK input could be valuable. The needs are communicated to the coordinating body at the King’s Centre for Global Health and THET and suitable healthcare professionals are identified to address the requirements.

## Methods

We contacted all 144 healthcare professionals who had volunteered for KTSP over the last 10 years with the exception of 13 for whom there was no current email address and invited them by email for an interview. Thirty-seven of these responded, agreeing to participate in the study. The criteria for inclusion in the study required participants to be healthcare professionals, who had visited Somaliland through KTSP as a volunteer and were working within the UK National Health Service (NHS). This excluded those who had worked on a consultancy basis and THET employees. Participants were excluded if they had visited Somaliland for the first time in the previous three months or if they were retired or did academic work only, as this would not allow for sufficient time or opportunity to evidence transferability of learning within the NHS. Twelve volunteers were excluded and 25 participants were eligible for interview.

The interview schedule was developed to generate in depth data on learning experience so we chose to conduct longer interviews with a smaller number of respondents. We selected a purposive sample of 12 to represent a range of different healthcare professional cadres; number of trips to Somaliland and purpose of trips, for example, teaching; student assessment and capacity building.

Ethical approval was gained from the King’s College London Biomedical Sciences, Dentistry, Medicine and Natural and Mathematical Sciences Research Ethics Subcommittee.

The interviews that lasted between one and two hours focused on examples of learning from the experience in Somaliland and its transferability to UK practice with participants encouraged to describe in detail up to five examples where they felt learning had occurred. Domains of both learning and transfer to work in the NHS were prompted using a framework adapted from a literature review on this topic [6] developed partly by the same team. Participants were prompted for learning on other topics, not mentioned in the framework, and for a description of the learning process and the barriers and facilitators to learning and transferability.

### Analysis

Interviews were fully transcribed and analysed using Nvivo 10. We extracted all descriptions of cases where the participants described learning and then used an iterative process of coding and review with refinement of coding strategies and re-coding. ET, PB and SHvW ensured consistency of coding and resolved differences through discussion. At least 20 % of all coding was cross checked by another researcher to ensure consistency of coding.

We used a framework describing the key domains of learning from health partnership work that we had developed from a systematic review of the literature [6] to analyse the interviews. Examples of learning experiences which participants described were classed according to these domains of learning, paying particular attention to any new domains that our study might add to this existing list. Each learning experience was then analysed for evidence of barriers and facilitators for learning and transferability to NHS practice. The case studies in Table 3 were specifically chosen to illustrate different levels of abstraction and adaptation that volunteers employed to transfer learning to the NHS.

## Results

Our sample of 12 volunteers included two mental health nurses, three midwives, one nurse, one paediatrician, one pharmacist, one psychiatrist, one psychotherapist and two surgeons. Four of the participants had taken part in one trip, four in two trips, two in three trips, and one in four trips and the last in over five trips. The purpose of the trips were predominantly teaching and training; two were for objective structured clinical exams (OSCEs) for nurses, two for supporting medical student exams, one for project scoping, and one for service capacity building. The participants, aged between 26 and 53 years old, had been in a healthcare role for between three and 35 years. Their daily activities included clinical, audit, financial management, people and project management, research, service development and teaching.

Each participant described between three and six examples of learning. All participants identified learning in communication and interdisciplinary working, teaching skills and personal development. We identified no new domains of learning other than those identified in the existing literature [6] and reflected in our interview schedule despite probing for additional areas of professional or personal development. All participants reported that at least one of their learning experiences could be transferred to their work within the NHS but not all learning was transferable to NHS practice. Table 1 shows the learning domains reported and where this was applicable to NHS work, with teaching and communication skills more reported as transferable more often than global health knowledge or research skills.

**Table 1:**
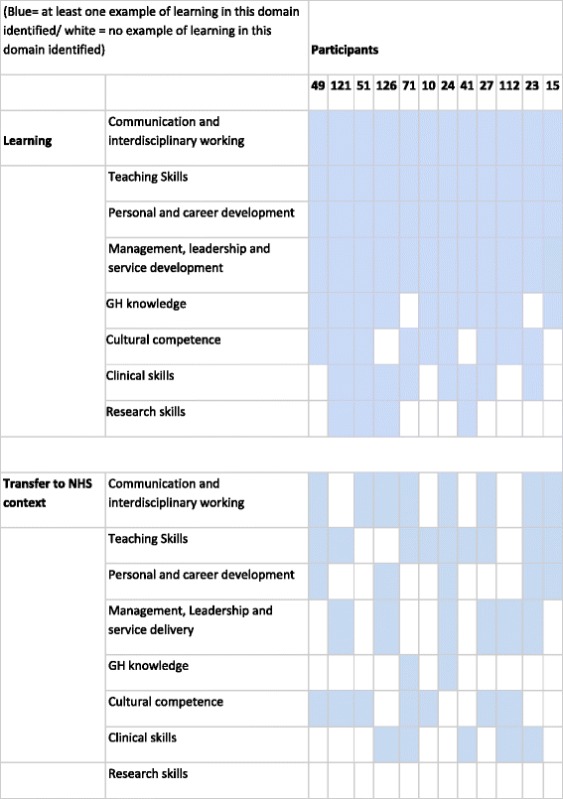
Learning domains and transfer to NHS work identified by participants

We coded all examples of learning according to the type of experience that participants reported as the source of learning to identify four types of experience that generated learning. Table 2 provides examples of learning within each category.

**Table 2:** Examples of learning

Experience	Learning	Translation to NHS context
Familiar practice in unfamiliar contexts		
A different approach to medical student learning. ‘*Somali system …. was a much flatter system, they were all pretty equal and they were sharing skills and obviously the more junior ones had more to learn than the more senior ones but it was very much a sharing insights and sharing skills.’*	Peer teaching as engaging and informative.	Encouraging junior doctor to student interaction for teaching on NHS ward rounds.
Alternative solutions to familiar problems		
Observing a midwife breastfeeding the baby of an acutely ill mother following post-partum haemorrhage when no formula is available. * ‘The mother was too unwell, she'd lost too much blood, and couldn't breastfeed or anything, so the midwife just took the baby and put the baby onto her breast, and fed the baby.’*	Alternative solution to a familiar problem. ‘*They don’t have formula…. thinking outside the box, it wasn’t acceptable here because everything would need to be screened and tested, yet probably saved that baby’s life at that point.’*	*‘Forcing you to think outside the box, to not be constrained necessarily by guidelines and protocols. I’m not saying that you should do it on the wards here, but feeling able to be open minded and broad minded, that that there are actually other ways around things.’*
Learning about Somali culture		
The role of relatives in the consultation.	Increased awareness of alternatives to the individual focus of consultations in UK culture to an appropriately family-centred approach	*‘You know sometimes you have to give the medication to the relatives and just keeping it at the back of my mind and thinking is it different the way we do it here or the way we do it in Somaliland it was a knowledge that I carried with me …. which one is better how can I apply, how can I improve you know.’*
Increased responsibility		
Leadership role on second trip to Somaliland despite being the least senior healthcare professional as they had previously been to Somaliland. *‘As a [younger person] managing people in their 40s or their 50s it’s not difficult, just you had to be able to talk to them in a way that they didn’t find you being patronising.’*	Leadership skills.	*‘I felt more confident to come back and my manager had been asking me to lead the ward for quite a while. And I felt more confident to come back and do that.’*
Interacting with senior government officials.	Skills to communicate interprofessionally with colleagues in more senior roles.	Improved professional communication skills and confidence.
New working relationships		
Working in an unfamiliar and sometimes stressful environment helped to facilitate new working relationships	Interprofessional support from UK colleagues.	Improved interpersonal relationships with colleagues from other disciplines when working in the NHS.
New working relationships with Somali colleagues ‘*Then in the afternoon, we’d go to the hotel, in the courtyard, to talk. And it was through talking and just social chats with the [Somali] lecturers.’*	Alternative healthcare professional cultures	Understanding a different professional culture

1. Familiar practices in unfamiliar contexts. Volunteers gained new knowledge by observing familiar healthcare delivery or health education in a different context. The new contexts included working across different professional, religious or community cultures and working in a resource-limited setting.
2. Alternative solutions to familiar problems. In some cases learning was triggered by observing a familiar problem with a very unfamiliar response, often related to working in a resource poor setting.
3. Experience of Somali culture. Several volunteers reported new knowledge of Somali culture. Some translated this to a better understanding of cultural diversity more generally, some limited this to an improved ability to communicate with Somali patients in their UK practice and others reported specific knowledge gained e.g. the rules on fasting for pregnant women.
4. Increased responsibility. Participants reported that increased responsibilities in their volunteering role in comparison with those in their NHS job increased confidence and encouraged personal and professional development.
5. Building new relationships: Participants learnt from new relationships with UK and Somaliland colleagues. These relationships cut across professional divides and NHS hierarchies and the two institutions within the partnership.

There was variation in the extent that volunteers reflected on their own responses to the experiences they identified as learning opportunities. This influenced both the learning from each experience and also its transferability to NHS practice. Cases 1, 2 and 3 [in Table 3] below illustrate this variability. In the first, the observation of familiar clinical practice in a context with less access to diagnostic technologies triggers reflection on technologies as a barrier to patient contact but also a facilitator in teaching good clinical practice within the NHS context. The volunteer reconsiders assumptions about the role of electronic measurement in clinical practice, gains new learning on what can be lost through electronic monitoring and changes her clinical practice and her teaching. In the second the volunteer reflects on the need to question assumptions about dress codes, rethinking her initial assumptions about the implications of the appearance of her Somali student. In the third the volunteer imports an idea of ‘African time’ from her UK experience and maintains this view during her time in Somaliland. The volunteering experience in this case does not challenge her to challenge the idea of a pan African approach to time keeping, culturally specific ideas about time in different parts of Africa or the potentially derogatory implications of her description.

**Table 3:** Case Studies

Case study 1	
Learning experience: Observing clinical practice in a context where diagnostic technologies are not available "It’s easy to just rely on a machine…… but there’s ways to improve on your practice. Go back to basics… Look at your patient. Touch your patient, talk to your patient”.	
Transfer to NHS: “When I came back to the UK …I wouldn’t reach for a machine, I would talk to the patient a lot, lot more. I would do a manual pulse rather than a machine pulse. I’ll say (to students) have you done the manual pulse? And they look at me like I’m crazy but …when you talk to them about the whys and all of that. You can pass on the knowledge that way.”	
Case study 2	
Learning experience: Observing a Somali student in a full burkha and concluding that “that person is really strict or somebody’s making them dress like that….then the student whipped it off and said I couldn’t be bothered to put any make up on today so I thought I’d wear my burkha.”	
Transfer to NHS: Using it in teaching. It is “good example of…you can make presumptions very easily, even if you think you are being kind or you think you are being open and then [I] use it as a very good example of just how similar people are the world over.”	
Case study 3	
Learning experience: Observing time keeping of staff in Somali Hospitals	
Transfer to NHS: “Large proportion of staff [in Kings College Hospital] are from Africa or the Caribbean. And it [the trip to Somaliland] made a big difference as to how I relate to the staff. I would ask for something and they wouldn't bring it straight away and I wouldn’t take it personally, because that’s part of their culture.”	
Case study 4	
Learning experience: Working with Somali patients	
Transfer to NHS: “Well it makes you more reflective in dealing with people from different cultures, with the Somali population it means you can immediately understand them so much more that before and so if, for example, there was a Somali patient in the hospital I’ll be asked to see them. Somali expert! Also, thinking about working with families, because in Somaliland there’s no such thing as the patient, it’s the patient and the family. So in the NHS, I always think that. It’s made me much more aware of carers and their role. And how it’s the patient and the carer. It perhaps, hasn’t helped me in terms of understanding people from other, like Sikh people or Hindu people or whatever. But the general framework of a cultural framework. It does make you reflect on those things and that does effect you in your day to things all the time.”	

### Factors influencing learning

Factors reported as influencing learning were the logistics of the trip (poor logistics distracted participants from learning); opportunities and support for reflection (long working days with little opportunity to debrief limited learning) and the extent to which participants were able to see links between their Somaliland experience and their UK work. In general, well-organised trips gave participants more time to focus on learning and supportive relationships with Somali partners, mentoring from UK colleagues or individual commitment to reflection, such as keeping a diary, facilitated learning.

### Factors influencing transfer of learning to NHS practice

Transfer of learning to NHS practice was influenced by individual volunteer factors, volunteer placement factors and volunteer NHS role. Individual factors included the extent of volunteer commitment or ability to extrapolate learning between contexts, placement factors included the links between roles while volunteering and within the NHS and NHS factors included organisational capacity for learning and change. Case study 4 below illustrates the translation of the volunteer experience to a broader appreciation of carers and their role in healthcare delivery and the inter-relation between culture and health. This volunteer effectively translates her learning to the NHS environment by extrapolating what they observe about the role of carers and adapting it the NHS context with reflection on the limits of her learning, that it, an acknowledgement that it may be culturally specific but also developing more generic learning about the role of carers in general.

## Discussion

This study reports data from interviews with volunteers from a long-term partnership between Kings Health Partners and organisations in Somaliland. It builds on previous work that documents learning outcomes from volunteering within healthcare partnerships [6, 7] through its focus on the process of learning from volunteer experience and the transferability of this learning to the UK NHS practice. Volunteers within KTSP reported learning in the domains of communication; interdisciplinary working; teaching; management; leadership and service development. As we identified no new domains of learning, we have not suggested any additions to the framework [6]. This learning came from observing familiar practices in unfamiliar environments; alternative solutions to familiar problems, experiencing Somali culture, opportunities to assume higher levels of responsibility and new professional relationships. There was variability in the extent to which volunteers were able to translate experience into learning and apply this learning to NHS practice. Time and support available for reflection and mentoring were important facilitators of this process.

Volunteering within links is underpinned by ideas of mutual learning [3] and there is an expectation that volunteers will gain new knowledge and skills from their involvement in healthcare partnerships. However, although healthcare partnerships often include formal training opportunities for healthcare professionals within the low or middle income partner organisations they may include little or no formal training for the UK based volunteers [1]. This reflects an expectation that learning from volunteering within partnerships will be learning from experience. Expectations of the learning outcomes from volunteering are often high and include reference to transformational learning where new experience encourages learners to reconsider pre-existing assumptions and to question and modify their frames of reference [8]. Educational theory on learning from experience suggests that it requires investment on the part of the learner and structures to support the learning process. Learning from experience requires critical reflection with the generation and testing of new ideas and revision of previous assumptions [8, 9]. This process is largely self-directed [8] but is facilitated by support to build the skills required for critical reflection, sufficient time for reflection and opportunities for discussion both with peers and with mentors [8, 10]

Volunteering in Somaliland stimulated questioning and reflection for all participants in our study. This learning came directly from experience of a different context. However, not all volunteer experiences generated learning. While some individuals reported an intensive process of questioning existing knowledge, generating new ideas and testing them to generate new knowledge others did not and this difference impacts on the professional development outcomes reported. Volunteers reported that well organised trips gave them more time to focus on learning and reflection. They reported that opportunities for discussion such as feedback from Somali partners and mentoring relationships added value to this.

Just as the process of experiential learning requires active engagement from the learner, the process of transferring learning to the NHS requires investment to translate learning in one context for use in another. All knowledge is to some extent contextual and requires modification for use in another setting [11]. Some is more easily transferable than others, for example technical skills can be more easily transferable than knowledge that arises from professional norms [11]. But in all cases volunteers needed to process and adapt learning to ensure transferability to NHS practice and valued acknowledgement of and support for this process.

Our interviews highlight the potential advantages of building learning and the application of that learning into the volunteer experience. This draws on current thinking on international service learning. Service learning is an educational strategy where students participate in an organised service activity that meets identified community needs and reflect on this experience with reference to a formal curriculum [12]. It has been mainly implemented as part of the curriculum within schools and higher education institutions and is often differentiated from volunteering that is not directly linked to a curriculum [13]. However it is a useful point of reference because it links service activity to a structured process of reflection that links it to the educational content of the course [12]. In volunteering within healthcare partnerships, there is no academic course with a pre-defined curriculum but there are professional development frameworks that identify areas of professional development for staff at all levels and in all disciplines within the NHS and work has been done to link these to volunteering outcomes [14]. Strategies such as personalised learning contracts with scope for modification and development during and after the placement could effectively structure volunteer learning processes and outcomes. Further research to assess the application of these strategies is required.

The discourse on partnership working proposes that UK volunteers are highly skilled professionals who can make a contribution to capacity building within low and middle income countries whilst bringing back new skills and attitudes from this experience. In practice, however, their role as teachers is sometimes emphasised over their role as learners. Partnership project outcomes, for example, focus on the impacts for low and middle income partners over those for UK partners [15]. Volunteer learning within partnerships is important for two reasons. Firstly, there is emerging evidence that the potential learning for volunteers could be significant in the Continuing Professional Development of UK health service staff, and secondly, it is integral to the idea of partnerships as two way learning processes. However, without framing volunteer learning as a primary outcome of health partnerships the learning is variable and dependent on the individual volunteer. This means that learning opportunities are often missed. We suggest that learning for UK health professionals should be explicitly built into the project plans of health partnerships and included in their monitoring and evaluation.

## Conclusion

We recommend that the process of learning from volunteering could benefit from a more overt framing of volunteering as a learning opportunity. Further research is required, particularly quantitative studies to measure the extent of the impact of volunteering on the professional development of clinicians. New tools for this purpose are available [16]. Achievement of learning outcomes should be supported with adequate time for reflection, access to relevant learning resources and mentoring. Acknowledgement of the need to actively translate and adapt learning for NHS contexts could increase the value of learning from volunteer placements for the UK healthcare workforce.

## Limitations

Not all volunteers who visited Somaliland could be contacted and those who are more committed to the partnership are more likely to have responded to the invitation to interview. Our sample may therefore exclude those who have had important negative experiences or those for whom the volunteering experience had little educational or other importance. Some of those interviewed were reflecting on volunteering experiences from several years previously and there may be inaccuracies or incompleteness in the recalled information.

## Abbreviations

KTSP: king’s thet somaliland partnership
NHS: national health service
OSCE: objective structured clinical examination
THET: tropical health and education trust
UK: United Kingdom
